# The role of adjuvant chemotherapy for patients with stage II and stage III gastric adenocarcinoma after surgery plus D2 lymph node dissection: a real-world observation

**DOI:** 10.1186/s40064-016-2552-3

**Published:** 2016-06-16

**Authors:** Chang-Fang Chiu, Horng-Ren Yang, Mei-Due Yang, Long-Bin Jeng, Aaron M. Sargeant, Su-Peng Yeh, Li-Yuan Bai

**Affiliations:** Division of Hematology and Oncology, Department of Internal Medicine, China Medical University Hospital, 2, Yude Road, Taichung, 40402 Taiwan; Cancer Center, China Medical University Hospital, Taichung, 40402 Taiwan; College of Medicine, School of Medicine, China Medical University, Taichung, 40402 Taiwan; Department of Surgery, China Medical University Hospital, Taichung, 40402 Taiwan; Charles River Laboratories, Preclinical Services, Spencerville, OH 45887 USA

**Keywords:** Gastric cancer, Adjuvant chemotherapy, Gastrectomy, D2 dissection, Relapse-free survival, Overall survival

## Abstract

**Background:**

The influence of adjuvant chemotherapy on the survival of gastric adenocarcinoma patients in a stage-specific manner is controversial.

**Methods:**

To further explore this topic, we retrospectively analyzed the impact of adjuvant chemotherapy on the clinical outcomes of 77 stage II and 117 stage III patients diagnosed between January 2008 and December 2012.

**Results:**

All 194 patients underwent radical operation plus D2 dissection, and were followed a median time of 23.3 (range 0.4–80.2) months. Median patient age was 67.7 (range 33.9–97.5) years. Adjuvant chemotherapy prolonged the relapse-free survival [22.9 (95 % confidence interval 9.4–36.4) vs. 14.2 (95 % CI 8.6–19.8) months, *P* = 0.009] and overall survival [32.3 (95 % CI 22.6–42.0) vs. 13.4 (95 % CI 9.5–17.2) months, *P* < 0.001] for patients with stage III, but not stage II, disease. Higher overall survival from adjuvant chemotherapy in stage II patients with node involvement did not reach the level of statistical significance (*P* = 0.102). To reduce the selection bias, 142 patients aged <75 years were included in a subgroup analysis in which the benefit of adjuvant chemotherapy on relapse-free survival and overall survival were demonstrated for patients with stage III disease.

**Conclusions:**

Adjuvant chemotherapy prolongs relapse-free and overall survival for patients with stage III gastric cancer in a real-world situation. Tailoring therapy based on different characteristics for patients with stage II gastric cancer may produce better outcomes.

## Background

Gastric cancer is the fourth most common cancer and second most common cause of cancer death globally, with a higher prevalence in East Asia (Hartgrink et al. 2009). The GLOBOCAN database from 1993 to 2001 showed that 933,293 patients were diagnosed with and 699,803 patients died of gastric cancer yearly (Kamangar et al. 2006). For patients diagnosed with non-metastatic gastric cancer, surgical resection has been the mainstay treatment modality.

People receiving operation for gastric cancer without adjuvant chemotherapy are at risk for disease recurrence. Results of the 15-year follow-up of the Dutch D1D2 trial suggested a 49 % recurrence rate after D1 lymph node dissection, and a 40 % recurrence rate after D2 lymph node dissection (Songun et al. 2010). In the study, more than 40 % of patients had stage I gastric cancer in either the D1 or D2 group. Patients with stage II gastric cancer accounted only for 24 and 23 %, and patients with stage III for 22 and 22 % in the D1 and D2 groups, respectively [staging according to American Joint Committee on Cancer (AJCC), 5th edition] (Fleming et al. 1997). In Eastern countries, D2 dissection is widely used post-operatively to reduce the recurrence rate. Data from a recent randomized trial enrolling only patients with II-IIIB gastric cancer revealed a 36 % recurrence rate in 5 years for patients without adjuvant chemotherapy after D2 dissection (Noh et al. 2014).

The value of adjuvant chemotherapy has been widely reviewed (Earle and Maroun 1999; Hejna et al. 2006; Fujitani 2013; Oh and Bang 2013). Before 2000, there was no large-scaled randomized phase III trial to evaluate the benefit of adjuvant chemotherapy on the survival of patients with gastric cancer after operation. Information on adjuvant chemotherapy in gastric cancer is largely derived from meta-analyses. Although some meta-analyses suggested a positive effect of adjuvant chemotherapy on survival of patients with gastric cancer (Hejna et al. 2006; Mari et al. 2000; Paoletti et al. 2010; Diaz-Nieto et al. 2013; Sun et al. 2009), no impact on survival had also been reported (Hermans et al. 1993). Even in meta-analyses with positive findings, a general agreement for adjuvant chemotherapy was not reached. Some studies suggested that adjuvant chemotherapy only be recommended for patients with positive lymph node (Earle and Maroun 1999), or not recommended because the benefit was small (Janunger et al. 2002). Fortunately, in the last decade, randomized, placebo-controlled, phase III clinical trials have proven the survival advantage conferred by adjuvant chemotherapy after D2 lymph node dissection (Noh et al. 2014; Sakuramoto et al. 2007; Sasako et al. 2011; Bang et al. 2012).

Although the value of adjuvant chemotherapy for gastric cancer is reaching general agreement by the results of phase III trials, there are few studies addressing the individual stage-specific benefit of adjuvant chemotherapy. The populations of patients and the inclusion criteria used in different studies were not the same (Sakuramoto et al. 2007; Bang et al. 2012; Nakajima et al. 2007). Additionally, the individual values of adjuvant chemotherapy for individual stage II or stage III were not the primary endpoints and thus were less addressed in the trials from the beginning. Although there were subgroup analyses, the benefits of adjuvant chemotherapy for stage II or stage III were not universal between trials. In the subgroup analysis of an S-1 adjuvant study, patients with stage II and IIIA disease had a 5-year relapse-free survival and a 5-year overall survival benefit from adjuvant S-1 (Sasako et al. 2011). However, only patients with stage II gastric cancer benefited from adjuvant chemotherapy with capecitabine plus oxaliplatin (XELOX) for a 5-year overall survival rate (Noh et al. 2014).

In this study, we reviewed the medical history of patients with stage II or stage III gastric cancer who received D2 lymph node dissection at our hospital (China Medical University Hospital) between January 2009 and December 2012. The relapse-free survival and overall survival of patients with adjuvant chemotherapy were compared to patients without adjuvant chemotherapy. The effect of adjuvant chemotherapy was further examined in a stage-specific manner.

## Patients and methods

### Patients

We performed a retrospective review of patients who were pathologically diagnosed as having stage II or stage III gastroesophageal junction (GEJ) or gastric adenocarcinoma after radical surgery and D2 lymph node dissection at China Medical University Hospital between January 2008 and December 2012. All information was obtained under a protocol approved by the China Medical University Hospital internal review board (CMUH105-REC1-047) in accordance with the ethical standards of the responsible committee on human experimentation and with the Helsinki Declaration of 1975. All pathologies were reviewed and reclassified according to the definition by AJCC 7th edition (Edge et al. 2010). Total gastrectomy was performed for patients with cancer of GEJ or upper gastric body, while subtotal gastrectomy was done for patients with cancer from lower body, antrum and pylorus. Patients with performance status of Eastern Cooperative Oncology Group (ECOG) 4 were excluded. In total, there were 77 patients with stage II gastric cancer and 117 patients with stage III gastric cancer. These patients were followed through October 2014.

### Adjuvant chemotherapy

According to the guidelines of our hospital, all patients with stage II or stage III gastric cancer were advised to receive adjuvant chemotherapy or concurrent chemoradiation. However, the final decision was made by the patient. The regimen of adjuvant chemotherapy for patients with stage II gastric cancer was a fluorouracil derivative (5-fluorouracil intravenously, UFT, capecitabine or S1 orally). For patients with stage III gastric cancer, the regimen of adjuvant chemotherapy was a combination of a platinum (cisplatin, carboplatin or oxaliplatin) and a fluorouracil derivative (5-fluorouracil intravenously or S1 orally). The adjuvant chemotherapy of intravenous medications (5-fluorouracil, cisplatin, carboplatin or oxaliplatin) will be given for 6 months, while oral fluorouracil derivative (UFT, capecitabine or S1) will be given for 12 months.

### Post-operative evaluation

All patients were followed and monitored according to the guidelines of our hospital. Patients receive esophagogastroduodenoscopic examination within 6–12 months after operation. In addition, patients received a computed tomography (CT) examination every 3–6 months for 2 years, followed by a CT examination every 6 months for 3 more years.

### Prognostic variables

To evaluate the effect of adjuvant chemotherapy on patient survival, clinical prognostic variables were collected for analysis, including gender, age, history of smoking, history of alcohol consumption, history of previous gastric operation for non-cancerous cause, performance status, preoperative carcinoembryonic antigen (CEA) value, preoperative CA19-9 value, and features of the pathology. The latter category included tumor size (T1, T2, T3 and T4), lymph node involvement (N0, N1, N2, N3), differentiation (well/moderate or poorly differentiated), and the presence or not of signet-ring cell morphology, lymphovascular permeation, perineural invasion, and *Helicobacter pylori*. CEA and CA19-9 were measured using a chemiluminescent immunoassay sandwich method (Beckman Coulter, CA). A positive *H. pylori* test was defined as either the presence of *H. pylori* organisms in tissue immunohistochemically or a positive Campylobacter-like organism test.

### Statistical analyses

The clinical characteristics of the groups (with or without adjuvant chemotherapy) were compared using a Chi square test for categorical variables and a Student’s *t* test for continuous variables. Relapse-free survival was the time interval between the gastrectomy date and the date of disease relapse. Overall survival was calculated from the diagnosis to date of death from any cause or the last follow-up date. Both the relapse-free survival curve and the overall survival curve were created using the Kaplan–Meier method. Statistical analysis was carried out using SPSS version 18 for Windows (IBM Corporation, Armonk, NY, USA) and SAS/JMP version 11 (SAS Institute Inc., Cary, NC, USA). Data are expressed as mean ± SD. All statistical tests were two-sided, and the differences were considered statistically significant at a P value <0.05.

## Results

### Patient baseline characteristics

Between January 2008 and December 2012, 194 patients were diagnosed as having stage II (n = 77) or stage III (n = 117) GEJ or gastric adenocarcinoma after radical operation plus D2 lymph node dissection at China Medical University Hospital. There were 125 male (64.4 %) and 69 female patients (35.6 %). The median age was 67.7 ± 12.7 (range 33.9–97.5) years. Six (3.1 %) patients had performance status ECOG 3 and 28 (14.4 %) patients had cancer from GEJ. Five patients died of acute complication after surgery (1 acute renal failure, 1 acute myocardial infarction, 1 massive bleeding, 1 pneumonia and 1 multiple organ failure).

For patients with stage II gastric cancer, 37 received adjuvant chemotherapy (9 intravenous 5-fluorouracil, 17 UFT, 11 S-1) while 40 did not. For stage III patients, 79 received adjuvant chemotherapy (8 intravenous 5-fluorouracil, 18 UFT, 4 capecitabine, 7 S-1, 23 intravenous 5-fluorouracil plus a platinum, 3 capecitabine plus a platinum, 16 concurrent chemoradiotherapy with intravenous 5-fluorouracil) while 38 did not.

**Fig. 1:**
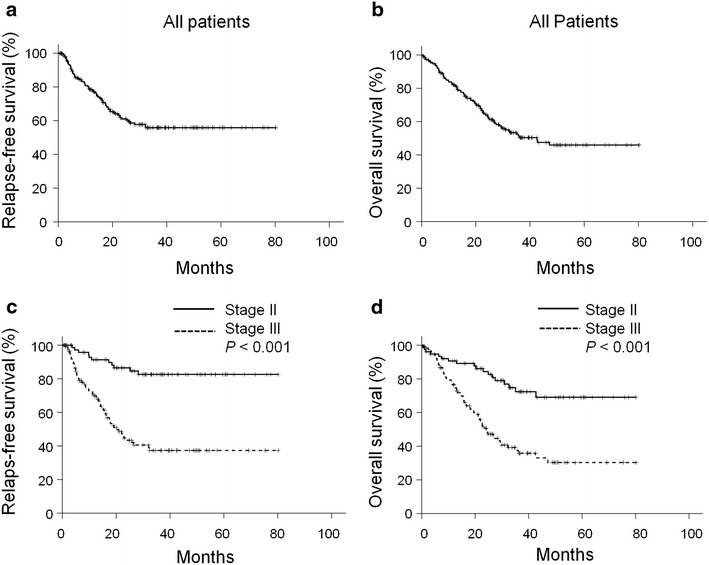
Kaplan–Meier curve of relapse-free survival and overall survival. **a**, **b** All patients (n = 194). **c**, **d** The survival curves are stratified by stage

With a median follow-up duration of 23.3 ± 18.6 (range 0.4–80.2) months, the median relapse-free survival was not reached (Fig. 1a), while the median overall survival time of the whole population was 42.6 months (Fig. 1b). For patients with stage II gastric cancer, both the median relapse-free survival and median overall survival time were not yet reached (Fig. 1c, d). For stage III patients, the median relapse-free survival and median overall survival time was 20.3 ± 2.6 (95 % CI 15.3–25.3) months (Fig. 1d) and 24.2 ± 2.4 (95 % CI 19.6–28.9) months (Fig. 1d), respectively. Stage was a significant prognostic factor for either progression-free survival or overall survival.

### Adjuvant chemotherapy prolongs relapse-free and overall survival for patients with stage III gastric cancer

**Fig. 2:**
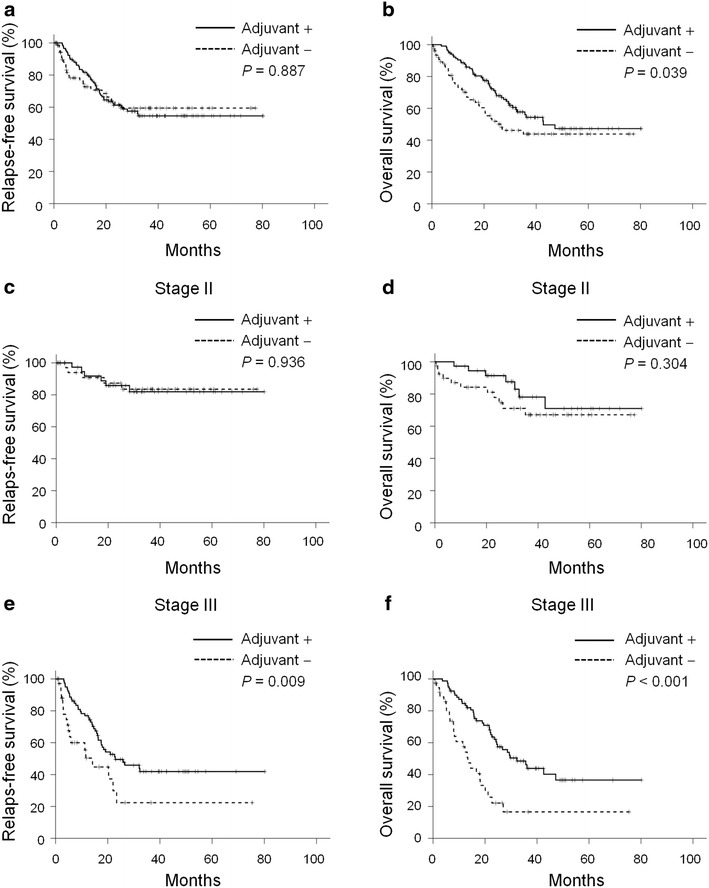
Kaplan–Meier curve of relapse-free survival and overall survival according to adjuvant chemotherapy. **a**, **b** All patients (n = 194). **c**, **d** Patients with stage II gastric cancer (n = 77). **e**, **f** Patients with stage III gastric cancer (n = 117)

The influence of adjuvant chemotherapy on relapse-free survival and overall survival was first examined in all patients with stage II or stage III disease. Although adjuvant chemotherapy did not benefit patients in terms of relapse-free survival (*P* = 0.887, Fig. 2a), it prolonged the overall survival time from 26.4 to 42.7 months (*P* = 0.039, Fig. 2b).

The significance of adjuvant chemotherapy was then evaluated for stage II (N = 77) and stage III (N = 117) patients separately. The adjuvant chemotherapy did not affect the relapse-free survival (*P* = 0.936, Fig. 2c) or overall survival (*P* = 0.304, Fig. 2d) for stage II patients. To the contrary, adjuvant chemotherapy prolonged both the relapse-free survival and overall survival for patients with stage III gastric cancer. The median relapse-free survival time was 22.9 ± 6.9 (95 % CI 9.4–36.4) months and 14.2 ± 2.9 (95 % CI 8.6–19.8) months for patients with or without adjuvant chemotherapy, respectively (*P* = 0.009, Fig. 2e). The median overall survival time was also prolonged in stage III patients to 32.3 ± 4.9 (95 % CI 22.6–42.0) months with adjuvant chemotherapy compared to 13.4 ± 2.0 (95 % CI 9.5–17.2) months without (*P* < 0.001, Fig. 2f).

### Stage II patients with positive node may benefit from adjuvant chemotherapy in overall survival

**Fig. 3:**
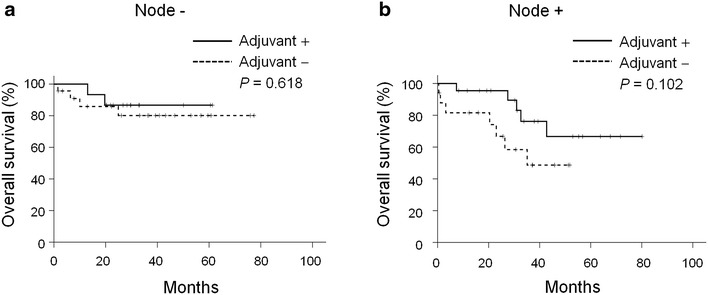
Kaplan–Meier overall survival curve of patients with stage II gastric cancer according to adjuvant chemotherapy. **a** Patients without lymph node involvement. **b** Patients with lymph node involvement

Because resectable gastric cancer patients with lymph node involvement have been reported to benefit more from adjuvant chemotherapy compared with patients without node involvement (Noh et al. 2014; Diaz-Nieto et al. 2013), we then evaluated the impact of adjuvant chemotherapy on the survival of stage II patients with or without node involvement (Fig. 3). Adjuvant chemotherapy did not affect the overall survival of patients without regional lymph node involvement (*P* = 0.618, Fig. 3a). Although there appeared to be a trend in adjuvant chemotherapy prolonging the overall survival of patients with lymph node involvement, the difference between these two populations was also not statistically significant (*P* = 0.102, Fig. 3b).

### Adjuvant chemotherapy prolongs relapse-free and overall survival for patients aged <75 years with stage III gastric cancer

**Fig. 4:**
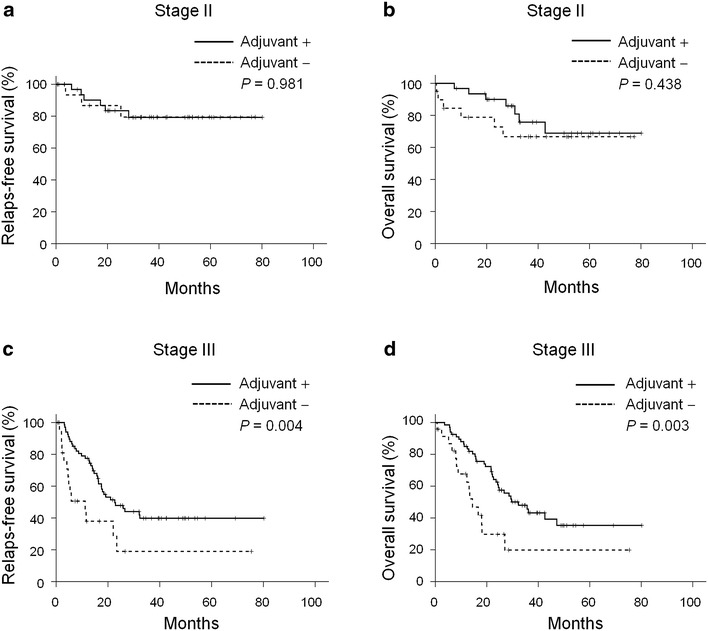
Kaplan–Meier curve of relapse-free survival and overall survival of patients aged <75 years according to adjuvant chemotherapy. **a**, **b** Patients with stage II gastric cancer (n = 51). **c**, **d** Patients with stage III gastric cancer (n = 91)

It is not surprising that elderly patients are less willing to accept chemotherapy than younger ones. To reduce the selection bias from age and take a closer look at the effect of adjuvant chemotherapy on survival, 142 patients aged <75 years were included in the survival analysis (Table 1). Ninety-eight patients received adjuvant chemotherapy while 44 did not. The baseline characteristic of patients in the two groups were similar except that patients in the adjuvant group had more lymph node involvement (*P* = 0.040). As shown in Fig. 4, adjuvant chemotherapy did not benefit the relapse-free survival (*P* = 0.981, Fig. 4a) or overall survival (*P* = 0.438, Fig. 4b) of stage II patients aged <75 years. For patients with stage III gastric cancer, adjuvant chemotherapy extended the median relapse-free survival from 11.2 ± 5.9 (95 % CI 0.0–22.7) months to 22.6 ± 4.4 (95 % CI 14.0–31.3) months (*P* = 0.004, Fig. 4c), and the median overall survival from 14.4 ± 2.5 (95 % CI 9.5–19.4) months to 32.3 ± 4.9 (95 % CI 22.7–41.9) months (*P* = 0.003, Fig. 4d).

**Table 1:** Baseline characteristics of stages II/III gastric cancer patients aged <75 years with or without adjuvant chemotherapy

	Adjuvant chemotherapy		*P* value
	With n (%)	Without n (%)	
Number of patients	98	44	
Age (year), median ± SD^a^	60.0 ± 9.3	64.7 ± 10.8	0.141
Gender, M/F	60/38	29/15	0.708
Smoking	38 (38.8)	17 (38.6)	1.000
Alcohol drinking	30 (30.6)	14 (31.8)	1.000
History of gastric operation	2 (2.0)	4 (9.1)	0.074
Location			0.371
GEJ	11 (11.2)	6 (13.6)	
Stomach	87 (88.8)	38 (86.4)	
PS			
0, 1	93 (94.9)	40 (90.9)	0.459
2, 3	5 (5.1)	4 (9.1)	
Preoperative CEA (ng/ml), median ± SD^a^	1.9 ± 6.8	2.3 ± 25.2	0.063
Preoperative CA19-9 (U/ml), median ± SD^a^	10.8 ± 88.7	7.4 ± 964.0	0.331
Tumor size			0.701
T1	4 (4.1)	0 (0.0)	
T2	8 (8.2)	4 (9.1)	
T3	49 (50.0)	26 (59.1)	
T4	37 (37.7)	14 (31.8)	
LN involvement			
N0	13 (13.3)	11 (25.0)	0.040
N1	10 (10.2)	9 (20.5)	
N2	34 (34.7)	6 (13.6)	
N3	41 (41.8)	18 (40.9)	
Differentiation			0.820
Well/moderate	20 (20.4)	9 (20.5)	
Poor	78 (79.6)	35 (79.5)	
Signet ring cell	43 (47.3)	21 (47.7)	0.705
Lymphovascular permeation	83 (84.7)	33 (75.0)	0.312
Perineural invasion	83 (84.7)	30 (68.2)	0.053
HP			0.467
Positive	41/72 (56.9)	10/21 (47.6)	
Negative	31/72 (43.1)	11/21 (52.4)	

*CEA* carcinoembryonic antigen, *GEJ* gastroesophageal junction, *HP* *Helicobacter pylori, F* female, *LN* lymph node, *M* male, *PS* performance status, *SD* standard deviation

Chi square test; ^a^ Student’s *t* test

## Discussion

Our study is a real-world observation of the benefit of adjuvant chemotherapy for patients with stages II and III GEJ or gastric adenocarcinoma. The characteristic of patients were not the same as those in clinical trials. For example, the median age of patients in our study was 67.7 years which was higher than the median age of 63 years and 56 years in the S1 trial and in the CLASSIC trial, respectively (Sakuramoto et al. 2007; Bang et al. 2012). However, our analysis confirms the benefit of adjuvant chemotherapy in patients with stages II/III gastric cancer outside a clinical trial. Furthermore, patients with stage III gastric cancer have a higher survival benefit from adjuvant chemotherapy than patients with stage II gastric cancer.

Although the value of adjuvant chemotherapy for gastric cancer is getting general agreement by evidence provided by phase III trials, there are still debates on several issues. First, the optimal regimens of adjuvant chemotherapy for gastric cancer are not yet clearly defined. The efficacy of S-1 (Sakuramoto et al. 2007; Sasako et al. 2011), UFT (Nakajima et al. 2007), XELOX (Noh et al. 2014; Bang et al. 2012), or ECX (epirubicin, cisplatin and capecitabine) (Cunningham et al. 2006) in adjuvant settings have been successfully demonstrated in individual phase III clinical trials. It is also unknown if combination chemotherapy is more effective than a single agent. Second, the populations of patients and the inclusion criteria used in different studies were not the same. In the UFT trial, only patients with T2N1-2 were enrolled into the trial (Nakajima et al. 2007). However, in the TS-1 trial, patients with stage II (excluding T1 cases), IIIA or IIIB were eligible for adjuvant therapy (Sakuramoto et al. 2007). In the CLASSIC trial, patients were eligible if they had stage II (T2N1, T1N2, T3N0), IIIA (T3N1, T2N2, T4N0) or IIIB (T3N2) gastric cancer (AJCC 6th) (Greene et al. 2002). Third, the individual values of adjuvant chemotherapy for stage II or stage III were not the primary endpoints and thus were less addressed in the trials at the beginning. Although there were subgroup analyses, the benefits of adjuvant chemotherapy for stage II or stage III are not universal in trials. In the subgroup analysis of the S-1 adjuvant study, patients with stages II and IIIA disease had a 5-year relapse-free survival and 5-year overall survival benefit from adjuvant S-1 in contrast with patients with stage IIIB disease (Sasako et al. 2011). In the follow-up report of the CLASSIC trial (adjuvant XELOX), either patients with stage II, stage IIIA or stage IIIB gastric cancer had a 5-year disease-free survival benefit from adjuvant XELOX. However, only patients with stage II gastric cancer benefited from adjuvant XELOX for a 5-year overall survival rate (Noh et al. 2014). Because of the high relapse rate for stage III gastric cancer and the insignificant benefit of S-1 for patients with stage IIIB disease in the subgroup analysis of adjuvant S-1 study (Sasako et al. 2011), the regimen of adjuvant chemotherapy at our institute was a fluorouracil derivative and a combination of a platinum and a fluorouracil derivative for patients with stages II and III gastric cancer, respectively.

The survival benefit of adjuvant therapy for patients with stage II only gastric cancer is rarely addressed (Chen et al. 2011). Although the subgroup analysis in either the TS-1 or CLASSIC trial suggested a positive effect of adjuvant chemotherapy for patients with stage II disease, controversial results have been reported (Chen et al. 2011; Coburn et al. 2008). Chen and colleagues retrospectively analyzed 268 patients with stage II gastric cancer who received adjuvant chemotherapy with either FAM regimen (5-fluorouracil, doxorubicin and mitomycin-C) or FOLFOX regimen (oxaliplatin, leucovorin and 5-fluorouracil) after D2 resection (Chen et al. 2011). The 1-, 3-, 5-, and 10-year overall survival rate was similar between patients who received adjuvant chemotherapy and those who did not. This uncertainty of adjuvant therapy for patients with stage II gastric cancer is also reflected by a retrospective analysis of the surveillance epidemiology and end results (SEER) database (Coburn et al. 2008). In this population-based analysis, adjuvant radiotherapy improved the overall survival of patients with stages III and IV M0 gastric cancer, but not for patients with stage IB and II gastric cancer (Coburn et al. 2008). In our study, the adjuvant chemotherapy did not benefit patients with stage II gastric cancer as well as those with stage III. There are a couple possible explanations. First, the adjuvant chemotherapeutic regimens for stage II gastric cancer used at our institute, which include UFT and intravenous 5-fluorouracil infusion without a platinum, are less potent than the XELOX agents used in the CLASSIC trial. Second, only 77 patients with stage II gastric cancer are included in our analysis.

Although the overall survival benefit of adjuvant chemotherapy was not realized for stage II patients, we found an apparent trend of the benefit for patients who had metastatic disease in lymph nodes (*P* = 0.102) compared with those who were node negative. The observation that patients with positive lymph node had more benefit from adjuvant chemotherapy has also been noted in the CLASSIC trial (Noh et al. 2014) and in the meta-analysis of the Cochrane Database (Diaz-Nieto et al. 2013). In the subgroup analysis of the CLASSIC trial, the adjuvant XELOX did not improve the overall survival for patients without node involvement (hazard ratio 0.79, 95 % CI 0.32–1.95), but did benefit for patients with lymph node involvement (hazard ratio 0.67, 95 % CI 0.51–0.87) (Noh et al. 2014). Similarly, the value of adjuvant chemotherapy on overall survival was demonstrated by a meta-analysis for patients with node involvement (hazard ratio 0.78, 95 % CI 0.67–0.91), but did not benefit patients without lymph node involvement (hazard ratio 0.74, 95 % CI 0.41–1.33) (Diaz-Nieto et al. 2013).

There are several caveats in our study that deserve further attention. First, this was a retrospective study conducted in a single institute. Second, the regimens of adjuvant chemotherapy used in our institute in the time period examined have now been replaced by newer or more intensive regimens in other countries. Since the reports of phase III trials, the use of adjuvant S-1 and XELOX has increased in recent years. However, both regimens are currently not reimbursed by the National Health Insurance in Taiwan. Therefore, chemotherapy with UFT, intravenous infusion of 5-fluorouracil with or without cisplatin, although less updated, is still widely used in adjuvant condition in Taiwan. Accordingly, our study reflects a real-world condition in which medical insurance determines the use of medication in most situations. Third, there were several kind of regimens used for adjuvant indication at our institute (see “Patient baseline characteristics” section). This heterogeneity of adjuvant regimen renders the analysis of influence of individual regimen on survival time impossible.

## Conclusions

In summary, our study provides evidence that adjuvant chemotherapy confer a relapse-free survival and an overall survival benefit for patients with stage III gastric cancer in a real-world situation. An apparent trend for an overall survival benefit also exists for stage II patients with positive lymph nodes. Tailoring therapy depending on different characteristics for patients with stage II gastric cancer may therefore produce better outcomes; however, further prospective trials are needed.

## Abbreviations

AJCC: American Joint Committee on Cancer
CEA: carcinoembryonic antigen
CI: confidence interval
CT: computed tomography
ECOG: Eastern Cooperative Oncology Group
GEJ: gastroesophageal junction
XELOX: capecitabine plus oxaliplatin
